# Myocardial iron intake following intravenous iron therapy with ferric carboxymaltose is sustained at 1 year despite recurrence of iron deficiency

**DOI:** 10.1111/bjh.19915

**Published:** 2024-11-19

**Authors:** S. K. Piechnik, P. Polzella, A. Shah, M. Vera‐Aviles, S. N. Kabir, M. Desborough, V. M. Ferreira, S. Lakhal‐Littleton

**Affiliations:** ^1^ Oxford Centre for Clinical Magnetic Resonance Research (OCMR) University of Oxford Oxford United Kingdom; ^2^ Department of Clinical Haematology Oxford University Hospitals NHS Foundation Trust Oxford United Kingdom; ^3^ Nuffield Department of Clinical Neurosciences University of Oxford Oxford United Kingdom; ^4^ Department of Physiology, Anatomy & Genetics University of Oxford Oxford United Kingdom

## Abstract

In clinical practice, intravenous (IV) iron therapy is used for the correction of iron deficiency. Patients with chronic causes of iron deficiency, for example, women with abnormal uterine bleeding, patients with inflammatory bowel disease often require repeated dosing with IV iron therapy. After a single standard dose of IV iron therapy (1000 mg) with ferric carboxymaltose, there is a rapid intake of iron into the myocardium, resulting in a sustained increase in myocardial iron content. The increase in myocardial iron content is independent of changes in plasma ferritin levels, and the recurrence of iron deficiency is not accompanied by a normalisation of myocardial iron. The most important implication is that repeated dosing with IV iron (ferric carboxymaltose) can result in cumulative build‐up of iron in the myocardium.

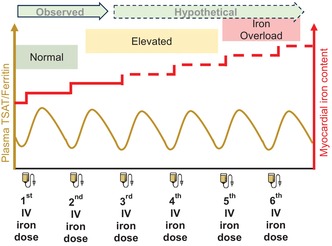

Dear Editor,

Intravenous iron therapy is now recommended in clinical guidelines for the management of iron deficiency (ID) across a number of specialties.^1^, ^2^, ^3^ While its efficacy in correcting ID and anaemia is well recognised, its impact on iron levels within organs remains poorly characterised. This knowledge gap is pertinent for two reasons. First, unlike most nutrients, iron cannot be excreted from the body, and any excess deposits in organs causing toxicity, as illustrated by the multi‐organ complications of haemochromatosis and β‐thalassaemia.^4^ Second, patients with chronic causes of ID, for example, abnormal uterine bleeding and inflammatory bowel disease, often require repeated doses, in the absence of any guidelines on what constitutes a safe maximal cumulative dose or any monitoring of organ iron levels. Iron levels within organs can be assessed non‐invasively by magnetic resonance T‐mapping, where a drop in T1 denotes an increase in organ's iron levels. Using this approach, we recently reported that a standard dose (1000 mg) of intravenous iron therapy with ferric carboxymaltose (FCM) in patients with ID raised myocardial iron within 3 hours, coinciding with full saturation of transferrin and a spike in non‐transferrin bound iron (NTBI).^5^ The rise in myocardial iron was sustained at 42 days despite the disappearance of NTBI, and declining transferrin saturation (Tsat) and ferritin levels.^5^ One question raised by these findings is whether the iron taken into the myocardium is eventually cleared.^6^ To address that question, we extended the study follow‐up to 12 months. Details of the clinical study and methods were as reported previously.^5^ Table 1 shows the demographics and baseline characteristics of the study participants. No participant had a history of cardiovascular and/or respiratory disease. None were tested for haemochromatosis HFE mutations.

**TABLE 1:** Demographics and clinical characteristics of all study participants.

Characteristics	All participants (*n* = 12)	Returned for 12‐month follow‐up (*n* = 8)
Age (years), median (IQR)	44 [27–69]	48 [40–48]
Sex, *n* (%)		
Female/Male	11 (91)/1 (9)^k^	7 (87)/1 (13)^k^
Weight (kg), median (IQR)	73.5 (56.5–77.0)	75 (55–82)
BMI (kg/m^2^), median (IQR)	27.2 (22.2–28.9)	27.5 (22.0–28.5)
Ethnicity, *n* (%)		
White	6 (50)^a,d,f,i,j,k^	4 (50)^a,f,i,k^
South Asian	4 (33)^b,e,g,h^	3 (37)^e,g,h^
Black	1 (17)^l^	0 (0)
Other	1 (17)^c^	1 (13)^c^
Reason for referral *n*, (%)		
Heavy menstrual bleeding	8 (66)^a,d,f,g,h,i,j,l^	5 (62)^a,f,g,h,i^
Abnormal uterine bleeding (e.g. fibroids)	1 (8.3)^e^	1 (13)^e^
Rectal bleeding	1 (8.3)^b^	0 (0)
IDA of unclear aetiology	2 (16.6)^c,k^	2 (25)^c,k^
Previous IV iron infusions, *n* (%)		
None	9 (75)^a,b,c,e,g,h,i,j,k^	7 (0)^a,c,e,g,h,i,k^
One prior infusion	3 (25)^d,f,l^	1 (13)^f^
Laboratory iron parameters at referral (normal range), median (IQR)		
Haemoglobin (120–170), g/L	105.5 (98.0–110.5)	106 (98–110)
Ferritin (10–300), mcg/L	7.4 (4.8–14.9)	7.4 (6.2–12.5)
Tsat (16–50), %	7 (5–8)	7.5 (7–8)

*Note*: Superscripts denote individual participants in (Table 1) and in (Figure 1).

Of the 12 participants invited, eight participants returned for follow‐up at 365 ± 3 days. Two had received a second dose of intravenous iron (FCM) as part of standard clinical care, in the interval between 42 days and 365 days. Participant ‘g’ received 500 mg iron at 205 days, while participant ‘i’ received 1000 mg iron at 320 days. The drop in myocardial T1 relative to pre‐infusion baseline was still significant at 365 days, whether second‐dose recipients were included (myocardial ∆T1 = −29.6 ms ± 24.6, *p* = 0.0234) or excluded (myocardial ∆T1 = −20.4 ± 17.3 ms, *p* = 0.0387) (Figure 1A). During the interval between 42 days and 365 days, the mean change in myocardial T1 in the six participants who had not received any further doses was +10.9 ± 16.9 ms, whereas it was −27.3 ± 20.3 ms in the two participants who had received a second dose.

**FIGURE 1:**
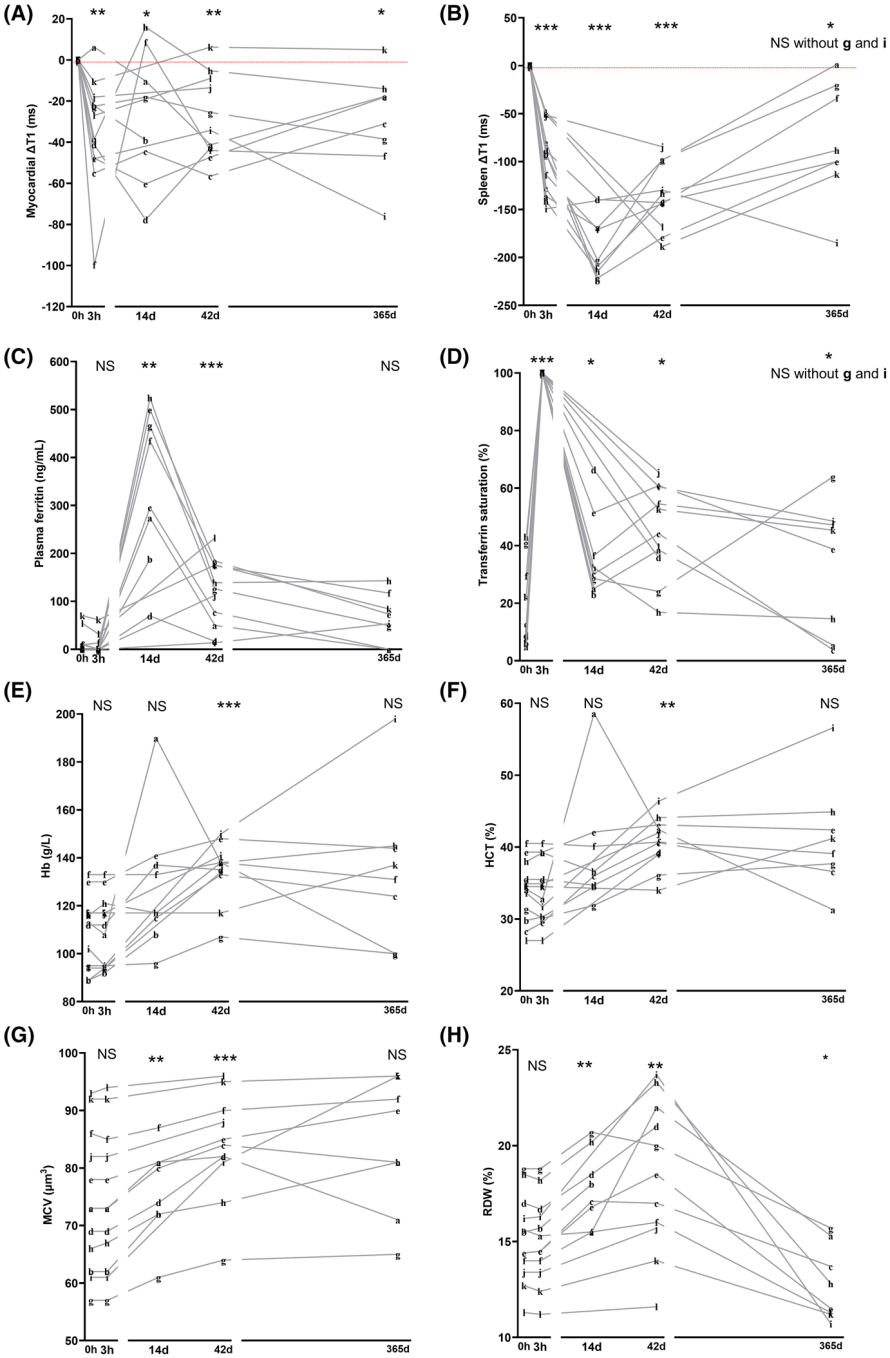
Myocardial iron intake following intravenous iron therapy with ferric carboxymaltose is sustained at 1 year despite recurrence of iron deficiency. Patients with ID and or anaemia received intravenous iron therapy with FCM (1000 mg iron) as part of standard care. They underwent MRI and blood sampling at baseline (0 h), then 3 hours (3 h), 14 days (14 d), 42 days (42 d) or 365 ± 3 days (365d) later. T1‐ mapping was used to assess changes in organ iron level relative to baseline. Negative ΔT1 values denote higher organ iron levels relative to pre‐infusion baseline. *p < 0.05, **p < 0.01, ***p < 0.001 relative to pre‐infusion baseline (0 h). Mixed effects analysis with Dunnett's test. (A) Changes in myocardial iron relative to baseline (myocardial ΔT1). (B) Changes in spleen iron relative to baseline (spleenΔT1). (C) Changes in plasma ferritin levels. (D) Changes in transferrin saturation (TSAT). (E) Changes in haemoglobin (Hb). (F) Changes in haematocrit (HCT). (G) Changes in mean corpuscular volume (MCV). (H) Changes in red blood cell distribution width (RDW). [Colour figure can be viewed at wileyonlinelibrary.com]

We also examined changes in spleen iron levels because spleen macrophages are considered a key intermediary in redistributing the iron from intravenous iron formulations.^7^ We had previously observed that the maximal drop in spleen T1 was at 14 days after FCM. At 365 days, the drop in spleen T1 relative to pre‐infusion baseline was only significant when second‐dose recipients were included (spleen ∆T1 = −79.8 ± 60 ms, *p* = 0.0143), (Figure 1B).

In terms of plasma iron markers, mean plasma ferritin at 365 days was no longer significantly different from pre‐infusion baseline (Figure 1C). Mean Tsat was only significantly higher than pre‐infusion baseline when second‐dose recipients were included (Tsat = 33.4% ± 22.5%, *p* = 0.041) (Figure 1D).

In terms of haematological parameters (Figure 1E–H), mean haemoglobin, haematocrit and mean corpuscular volume were no longer significantly different from baselines, whether second‐dose recipients were included or not. Red cell distribution width remained significantly lower than pre‐infusion baseline whether second‐dose recipients were included or not.

Of the eight participants who returned at 365 ± 3 days, six (including participants ‘g’ and ‘i’) now had ferritin <100 μg/L, three had Tsat <20% and two (including participant ‘g’) were anaemic.

The central finding of this study is that myocardial iron intake following a single standard dose of intravenous FCM is sustained for at least 1 year. In contrast, spleen iron declined over this period. Uncoupling of changes in myocardial from spleen iron is consistent with our previous report that the myocardial iron intake is not via the intermediary of spleen macrophages.

Another key finding is that the rise in myocardial iron is sustained despite the decline in plasma Tsat, ferritin and haematological parameters. This demonstrates that the recurrence of ID or of anaemia is not necessarily accompanied by a normalisation of myocardial iron levels.

The most significant implication of these findings is that repeated infusion with FCM may cumulatively build‐up myocardial iron levels. Consistent with this, we observed a further rise in myocardial iron in the two participants who received a second dose. Extrapolating from the effect of a single 1000 mg iron dose, we estimate that six standard doses would lower myocardial T1 to below the 850 ms cut‐off, a strong CMR‐based predictor of iron overload cardiomyopathy in patients with β‐thalassaemia.^8^ However, a larger study is needed to formally examine cumulative build‐up, and to identify the determinants of long‐term myocardial iron retention, in patients on long‐term intravenous iron therapy.

Another important implication is that ferritin and Tsat thresholds are not suitable for safeguarding against the risk of myocardial iron overload, as the levels of these iron markers are uncoupled from myocardial iron intake.

Our findings show that T1‐mapping is useful for monitoring changes in myocardial iron in otherwise healthy individuals. However, its utility maybe more limited in certain patient groups with confounding factors, for example, interstitial fibrosis.

Current guidelines do not define a safe maximal cumulative dose of intravenous iron. Data on the safety of long‐term intravenous iron therapy for the heart remain limited, with some studies reporting no adverse effects and lower mortality and others reporting increased mortality.^9^, ^10^, ^11^, ^12^, ^13^, ^14^ It remains unclear if these safety data can be extrapolated to real‐world settings. For instance, the actual cumulative doses delivered were rarely reported, myocardial iron and heart function were not actively monitored, and the length of follow‐up, typically 6 months to 2 years, may not have been sufficient for any cardiotoxicity to manifest clinically.

Our findings highlight the urgent need for studies that examine changes in myocardial iron and heart function in patients on long‐term intravenous iron therapy, to ensure the benefits of this therapy continue to outweigh any potential harms.

## AUTHOR CONTRIBUTIONS

S.L.‐L. conceived project, secured funds, analysed data and wrote the manuscript. P.P. and M.D. recruited patients. A.S. performed study procedures in participants. V.M.F. and S.K.P. designed MR imaging protocol and analysed MR data. M.V.‐A. and S.N.K. performed experiments and analysed data. All the authors reviewed and commented on the manuscript.

## FUNDING INFORMATION

S.L.‐L., M.V.‐A. and S.N.K. were funded by a Medical Research Council Senior Research Fellowship awarded to S.L.‐L. (MR/V009567/1/) and the British Heart Foundation Centre for Research Excellence (HSR00031 and RE/18/3/34214). A.S. was funded by a National Institute for Health and Care Research (NIHR) Academic Clinical Lectureship award.

## CONFLICT OF INTEREST STATEMENT

S.L.‐L. reports receipt of previous research funding from Vifor Pharma, personal honoraria on a lecture from Pharmacosmos and consultancy fees from Disc Medicine and ScholarRock. A.S. is an Editor of Anaesthesia.

## ETHICAL APPROVAL

The Study of Tissue Iron Uptake in iron‐deficient patients following IV iron therapy (STUDY) is an investigator‐initiated, prospective, observational study conducted at one UK site, sponsored by the University of Oxford. The trial protocol and amendments were approved by a NHS Ethics Committee in the UK (North West—Liverpool Central Research Ethics Committee Ref: 22/NW/0172), and the Health Research Authority.

## PATIENT CONSENT STATEMENT

Patients provided written informed consent.

## CLINICAL TRIAL REGISTRATION (INCLUDING TRIAL NUMBER)

The study was registered prospectively on the ISRCTN registry (ISRCTN15770553) and ClinicalTrials.gov (NCT05609318).

## Data Availability

Data will be made available upon reasonable request to the senior author.
